# Effectiveness of low frequency vibration on the rate of canine retraction: a randomized controlled clinical trial

**DOI:** 10.1038/s41598-024-58268-4

**Published:** 2024-04-04

**Authors:** Mohamed Atfy Abd ElMotaleb, Amr Ragab El-Beialy, Fouad Aly El-Sharaby, Amr Emad ElDakroury, Ahmed Abdelsalam Eid

**Affiliations:** Orthodontic Department, Faculty Dentistry, Cairo University, 11 ElSaraya St. Manial, Cairo, Egypt

**Keywords:** AcceleDent, Acceleration of tooth movement, Vibrating devices, Canine retraction, Digital models, Three-dimensional imaging, Randomized controlled trials

## Abstract

To investigate the effectiveness of AcceleDent Aura vibrating device on the rate of canine retraction. Thirty-two patients requiring extraction of upper first premolars and canine retraction were randomly allocated with a 1:1 ratio into either no-appliance group or the AcceleDent Aura appliance group. Canine retraction was done applying 150gm of retraction force using NiTi coil springs on 16 × 22 stainless steel archwires. The duration of the study was 4 months. Models were collected and digitized directly after extraction of upper first premolars and at monthly intervals during canine retraction for recording the monthly as well as the total distance moved by the canine. Digitized models were superimposed on the initial model and data were statistically analyzed. Anchorage loss, rotation, tipping, torque and root condition were evaluated using cone beam computed tomography imaging. Pain was evaluated by visual analog scale. No patients were dropped-out during this study. There was no statistically significant difference between both groups regarding the total distance travelled by the canine (*P* = 0.436), as well as the rate of canine retraction per month (*P* = 0.17). Root condition was the same for the two groups. Regarding the pain level, there was no statistically significant difference between the two groups at day 0 (*P* = 0.721), after 24 h (*P* = 0.882), after 72 h (*P* = 0.378) and after 7 days (*P* = 0.964). AcceleDent Aura was not able to accelerate orthodontic tooth movement. Pain level couldn’t be reduced by vibrational force with an AcceleDent device during orthodontic treatment. Root condition was not affected by the vibrational forces.

## Introduction

Shortening the treatment time via accelerating orthodontic tooth movement (OTM) continues to be a relentless challenge and demand for orthodontists, patients and even parents^1,2^ The prolonged orthodontic treatment time bares many risks among which are the decalcifications, caries, gingivitis, periodontal breakdown, possible root resorption and have a greater negative impact on the quality of life and facial esthetics of patients^3–7^ To date, several modalities have been investigated to accelerate OTM including the invasive and the minimally invasive surgical techniques (dentoalveolar distraction, alveolar surgeries to undermine interseptal bone, and alveolar corticotomies), the non-invasive device-assisted techniques including cyclic vibrations, Low-Level Laser therapy (Photobiomodulation), direct light electric current, static or pulsed magnetic field and the systemic and local administration of biological substances; such as hormones and medications^1,8^

Each of the aforementioned approaches for accelerating orthodontic tooth movement has its own limitations and drawbacks^7^ The surgical approaches are invasive, associated with increased morbidity related to the technique, limited-time efficiency, with some risk of root damage, expensive, limited clinical evidence, are accompanied by post-operative pain and swelling, and hence less patient acceptance^1,3,4,8^ The low level laser energy and the pulsed electromagnetic fields can cause local pain, root resorption^4^ and need specialized costly equipment. The pulsed electromagnetic fields could adversely affect protein metabolism and muscle activity, while the direct current could cause a tissue- damaging ionic reaction^3^ The pharmacological techniques bare the risks of local pain and root resorption^9^ The local injections of prostaglandins, vitamin D3, and osteocalcin are painful, with patient discomfort and could illicit a detrimental inflammatory response^6^ The application of these biologic compounds could become standard practice in clinical orthodontics in the future, but more evidence is needed to evaluate their safety, efficacy, and specificity to the dentoalveolar tissues^1^ This leaves the vibrating devices as the non-invasive, most palatable and user-easy-to-use example of physical acceleration of OTM^4,10^

AcceleDent vibrating device (OrthoAccel Technologies, Houston, Texas) has been introduced to the market in 2009. It was intended to be used by the patients in conjunction with fixed orthodontic appliances or aligners, for 20 minutes per day by gently biting on the vibrating plastic wafer. It vibrates at a frequency of 30Hz and has a force amplitude of 20 grams. The mechanism of action in humans and animal studies is hypothesized to be via enhancing bone remodelling through increased RANKL expression together with elevation in IL-1 beta levels^6,11–15^ with a result in rise of the rate of tooth movement. The results of the randomized controlled trial and the systematic reviews reported in the literature concerning the efficiency of these devices in acceleration of tooth movement are controversial^4,6,11,16–19^.

The aim of this study is to investigate the effectiveness of AcceleDent Aura vibrating device on the rate of canine retraction. The null hypothesis was set as no difference in the rate of canine retraction between AcceleDent Aura appliance group and no appliance group. Other side effects including pain and root resorption were also considered.

## Results

**Figure 1:**
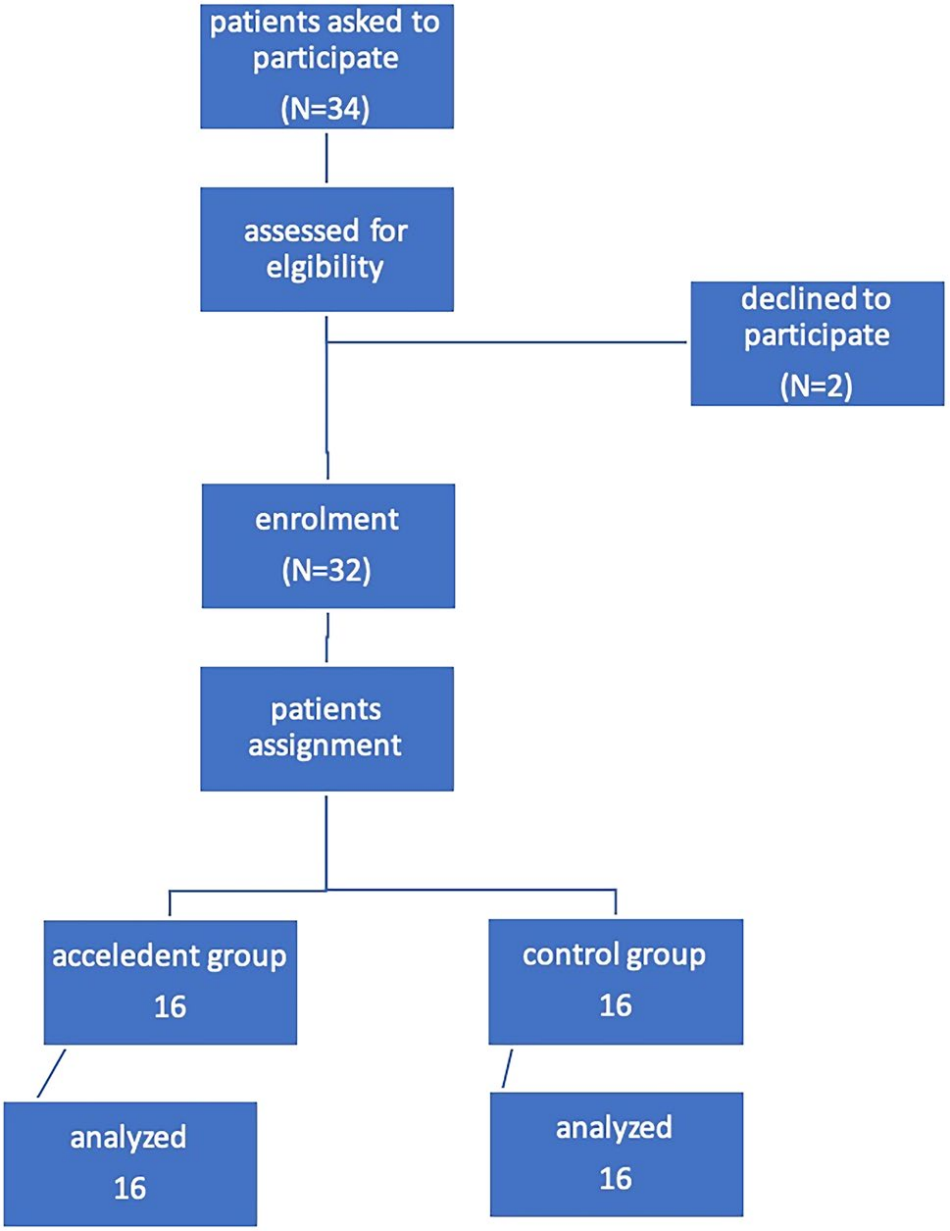
Consort diagram showing the flow of subjects through the study.

A CONSORT chart showing participant flow during the current study (Figure 1). The baseline characteristic values showed homogenous criteria between both intervention and control groups. In the AcceleDent group, the mean distances moved by the canine in the 1st, 2nd, 3rd and 4th months were (1.5° ± 0.8°), (1.5° ± 0.8°), (1.3° ± 0.7°) and (1.1° ± 0.6°) respectively compared to (1.3° ± 0.7°), (1.6° ± 1.3°), (0.9° ± 0.7°) and (1.3° ± 1.1°) respectively in the no-appliance group with no statistically significant differences except at the 3rd month (*P *= 0.009) (Table 1).

**Table 1:** Comparison of the mean differences in the amount of canine retraction for each month between both groups.

Measurement		N	Mean	SD	Mean difference	SED	95%CI—Lower	95%CI—Upper	t	df	*P*-value
1st month	Intervention	32	1.48	0.83	0.17	0.19	− 0.21	0.56	0.89	62	0.376
	Control	32	1.31	0.71							
2nd month	Intervention	32	1.48	0.84	− 0.16	0.27	− 0.70	0.37	− 0.61	62	0.542
	Control	32	1.64	1.26							
3rd month	Intervention	32	1.33	0.73	0.47	0.17	0.12	0.81	2.70	62	0.009*
	Control	32	0.86	0.66							
4th month	Intervention	32	1.09	0.61	− 0.22	0.22	− 0.65	0.21	− 1.02	62	0.313
	Control	32	1.31	1.06							

*significant at *p* ≤ 0.05.

The total distance moved by the canine as measured on the digital models was (5.4 ± 1.2 mm) and (5.1 ± 1.4 mm) for the AcceleDent and the no-appliance group, respectively with no statistically significant difference reported (*P *= 0.436) (Table 2). Regarding the total distance moved by the canine as measured on CBCT, there was no statistically significant difference between two groups, where the canine tip moved in intervention and control groups by 4.8 mm and 4.5 mm respectively. In the intervention group, the mean distance moved by the canine cusp tip, center and apex as measured to the frontal plane were (4.8 ± 1.3), (1.9 ± 0.8) and (0.1 ± 1.3) respectively, while in the control group, these distances were (4.5 ± 2.3) (1.7 ± 1.5) and (0.4 ± 1.0) respectively (Table 3).

**Table 2:** Mean, standard deviation and the result of for comparison of the total distance moved by the canine as between the two groups measured on the digital models using independent t test.

	Intervention	Control	*P-*Value
Mean	5.38	5.1	0.436
SD	1.2	1.4	

Significance level*: *P* < 0.05.

**Table 3:** The distances moved by the canine cusp tip, center and apex for the two groups measured on CBCT.

Measurement		N	Mean	SD	Mean difference	SED	95%CI—Lower	95%CI—Upper	t	df	*P*-value
Distance Canine cusp-FP	Intetrvention	32	4.8	1.3	0.22	0.47	− 0.72	1.16	0.46	62	0.645
	Control	32	4.5	2.3							
Distance Canine center—FP	Intetrvention	32	1.9	0.8	0.14	0.29	− 0.45	0.73	0.48	62	0.632
	Control	32	1.7	1.5							
Distance Canine apex-FP	Intetrvention	32	0.1	1.3	− 0.29	0.29	− 0.87	0.30	− 0.98	62	0.33
	Control	32	0.4	1							

The two groups were similar for canine tipping (Table 4), rotation (Table 5), and root resorption (Table 6). No statistical difference between the groups was reported for pain intensity (Table 7).

**Table 4:** Mean, SD and the result of Kruskal Wallis test for comparing the canine tipping between the two groups.

Tipping		Intervention	Control	*P-*Value
Canine-HP	Mean	10.2	11.3	0.337
	SD	4.1	5.5	
Canine-FP	Mean	− 10.1	− 8.9	0.452
	SD	4.1	14.2

**Table 5:** Mean, standard deviation (SD) and result of independent *t* test for comparison of canine rotation between the two groups.

Rotation		Intervention	Control	*P-*Value
Canine-MSP	Mean	12.0	15.1	0.172
	SD	7.9	10.0	
Canine-FP	Mean	− 11.4	− 15.0	0.146
	SD	8.9	10.5

**Table 6:** Mean, standard deviation (SD) and result of independent *t* test for comparison of the change in canine length between the two groups.

Canine Length	N	Mean	SD	Mean difference	SED	95%CI—Lower	95%CI—Upper	t	df	*P*-value
Intervention	32	0.8	0.7	0.24	0.22	− 0.21	0.68	1.07	62.00	0.289
Control	32	0.6	1

**Table 7:** Median, minimum, maximum values and result of Kruskal–Wallis test for comparison of the pain intensity after starting canine retraction in the two groups.

Timing		Intervention	Control	*P-*Value
At same day	Median	4	5	0.721
	Min	0	0	
	Max	10	8	
24 h	Median	4	5	0.882
	Min	0	1	
	Max	10	8	
72 h	Median	2	3	0.378
	Min	0	1	
	Max	8	5	
7 days	Median	0	0	0.964
	Min	0	0	
	Max	4	2	

## Discussion

The orthodontic evidence reports that the average time for orthodontic treatment is 24 months^2,3,12,19,20^ This is considered to be an extensive period for the patients to maintain compliance with oral hygiene measures and agreement to appointments. Uribe et al.^1^ concluded that orthodontists, patients as-well-as guardians did not favor invasive approaches for reducing orthodontic treatment time. The non-surgical mechanical or physical approaches for accelerating OTM have gained great popularity in the orthodontic field due to their non-invasive nature, and ease of use either by the clinicians or the patients.

Among the most recent non-invasive approaches for accelerating OTM is the vibrating devices. Acceledent Aura is one of the recent devices that produce gentle vibration micropulses (0.25 N at 30 Hz). It is claimed that this amount of vibration increase the RANKL expression together with elevation in IL-1 beta levels which enhances the bone remodelling^6,11–15,21^, with a result in rise of the rate of tooth movement.

A number of authors; Nishimura et al.^6^, Leethanakul et al.^13^, Alikhani et al.^14^, Gujar et al.^22^, Judex and Pongkitwitoon^23^ and Pavlin et al.^9^; who worked on invitro, experimental and on human samples reported a correlation between the vibrational forces and the increased signaling pathways and inflammatory mediators such as NF kappa-B and ligand (RANK/RANKL) expression, IL-1β secretion in the gingival crevicular fluid and osteoclastic activation with a conclusion that it might increase OTM. On the contrary, multiple other authors; Miles et al.^16,24,25^, Woodhouse et al.^17^, Idarrag et al.^10^, Yadav et al. ^26^, Dibiasae et al.^27^, failed to find any effect of vibrational forces on the speed of orthodontic tooth movement. Uribe et al.^19^ found conflicting results in animal and human studies. Extreme findings were also reported in the literature where kalajzic et al.^28^ found a significant decelerating effect on OTM with the use of vibrational devices.

Although the abovementioned studies that measured the effect of vibrational forces on accelerating OTM ranged between animal, invitro, biochemical markers and human studies, with different measured parameters, the contradicting findings are striking. The only consensus of the narrative and systematic reviews^4,7,19,29,30^, is that there is insufficient evidence, and the available evidence is of low quality. They reported that the conducted research is questionable accompanied by many flaws, with unclear or high risks of bias, and sometime the journal was not peer reviewed. They recommended that further well-designed, properly conducted and rigorous randomized controlled trials are needed to determine whether vibrational forces may result in a clinically important reduction in the duration of orthodontic treatment, without any adverse effects. A fact that provoked the execution of the current study to investigate the effectiveness of AcceleDent Aura vibrating device on the rate of canine retraction.

The current study was designed as an randomized controlled trial to investigate the effect of AcceleDent Aura appliance (30 Hz, 0.2 N or 25 g) used by the patients for 20 min daily for canine retraction using two comparable groups; the active group and the control group. The mechanics utilized are the everyday conventional mechanics used to retract the canine using conventional coil spring delivering 150 gm of force. We believe this applied mechanics are acceptable to represent the rate of canine retraction using conventional mechanics. The applied method of retraction was matched other with the trials which used the same retraction method Miles et al.^25^, Wagh et al.^2^ and Pavlin et al.^9^, but was totally different from the mechanics utilized by Nishimura et al.^6^ Nishimura et al.^6^ used transpalatal expansion spring. They measured the accelerated OTM in the first order (buccolingually) rather than in the second order (mesiodistally). This force represents some skelet al. as well as dental expansion, which comprises the majority of the actual OTM and undermines the results.

According to Shpack et al.^31^, the time needed for the contact between the canine and second premolar is 4 months, which was the time set for the current study, resembling the study duration of DiBiase et al. ^27^, and Idarraga et al.^10^, but longer than that the 10 weeks study duration reported by Miles et al.^16,24^ and the 3 months study by Leethanakul et al.^13^. This duration was elected because an early space closure would affect the overall mean rate of movement for the whole sample and hence disrupt the statistical analysis. Besides, the logical explained by Miles et al.^25^ that compliance with the vibrational devices might fade over time if an extended experimental time is tested.

In the study by Wagh et al.^2^, they used the orthopantomogram (OPG) to evaluate the root resorption irrespective of its inherent flaws of being a 2D image with superimposition and distortion errors. The current study used CBCT to assess the effect of cyclic vibrational forces on the OTM, angulation and root length. This was in accordance the study by Kau et al.^11^.

For measurement of the OTM, former studies^13,16,24,25,27^, used the physical plaster models to evaluate the rate of OTM under investigation. This modality of measurement represented a point of weakness for orientation of models on the same plane. Using the occlusal plane as the reference plane was subjected to changes as a result of continuous tooth movement^32,33^. Uncommonly, Pavlin et al.^9^ measured the rate of canine retraction directly in the patient mouth by calculating the distance between the canine cusp tip and temporary anchorage devices (TADs). This is a true accountable source of error, because it was reported that directly or indirectly loaded TADs are a potentially unstable landmarks. TADs do not remain absolutely stationery and that they might show some change of angulation during orthodontic loading although they are still anchored to the bone^34,35^. Thus, using the TADs as reference points for measuring the amount of canine retraction is highly questionable, and might not be a true indicator of space closure^25^. Rossini et al.^36^, and Sakar et al.^37^ proved that digital models have 1:1 ratio to physical reality, and suggested that they could be considered as the new gold standard in current orthodontic research and practice. Woodhouse^17^ and wagh^2^ measurements were done on digital models. The current study is the first encounter in the literature to use CBCT together with digitized 3D models in a randomized controlled trial for accurate assessment of the rate of canine retraction using acceleration vibrational devices.

Upon comparing the rate of canine retraction between the experimental group and the control group, it was found that the total distance travelled in 4 months was 5.4 ± 1.2 mm in the experimental group and 5.1 ± 1.4 mm in the control group. The mean distances moved by the canine in the 1st, 2nd, 3rd and 4th months were (1.5° ± 0.8°), (1.5° ± 0.8°), (1.3° ± 0.7°) and (1.1° ± 0.6°) respectively in the experimental group, while in the control group they were (1.3° ± 0.7°), (1.6° ± 1.3°), (0.9° ± 0.7°) and (1.3° ± 1.1°) respectively. These differences were neither statistically nor clinically significant. The rate of OTM reported in the current study lies within the logical, scientifically known rate of OTM/month. These same findings were similar to that mentioned by Woodhouse et al.^17^, Miles and Fischer^24^, Miles et al.^16,25^. The rate of the canine retraction in the current study was higher than that reported by Pavlin et al.^9^ in their experimental and control groups which was (1.16 mm/months and 0.79 mm/month respectively), but less than that reported by Leethanakul et al.^13^ who found rate of canine retraction in experimental group to be 2.85 mm.

Miles and Fisher^24^, Kau et al.^11^ measured the change in the anterior arch perimeter and irregularity index during the initial alignment in the mandibular arch. Although the measured parameter seems logical, it measured the OTM in the labial direction. In our study, we measured the canine retraction phase which is the phase that consumes a considerable amount of the treatment time.

Gujar et al.^22^, and Wagh et al.^2^ measured the rate of canine retraction through measuring the closure of the extraction space using a vernier caliper. This method of measurement does not take into account the loss of anchorage of the molar which might have affected the results.

Our current study was self-funded to eliminate any source of bias such as that reported in the Dibiase et al.^27^ study, where both the functional and sham AcceleDent^®^ units were donated by the OrthoAccel Technologies Inc, (Bellaire, Tex, USA). In contrast to our study, Pavlin et al.^9^ reported a significant increase in the OTM using the vibrational device. However, their research results were questionable because their research was supported by a grant from OrthoAccel Technologies (Bellaire, Tex), their leading author was a consultant for OrthoAccel, and the publishing journal is not peer reviewed^4^.

AcceleDent Aura is a device that depends on the patient’s compliance, which is real research challenge. In the current study, the authors tried to increase the patients compliance by informing them that their devices contained a memory chip that store the data of their usage to the device and that it will be collected as part of the trial and they were asked to write down the number of minutes of usage in a logbook. In the current study, the subject’s compliance was about 89%. However, nothing can prevent the patients from turning on the appliance without putting it in their mouth. In the current study the canine tipping, torque and rotation were similar between the two methods.

Results of the current study nullified the effect of vibration on orthodontically induced root resorption. These same results were reported by kau^12^, DiBiase et al.^27^, and Wagh et al.^2^. In this study it was found that AcceleDent has no effect in reducing pain score, which is similar to the results by Woodhouse et al.^17^ and Miles et al.^16^ and Miles and Fisher et al.^24^,

To sum up, the results of the current study provides evidence that the vibrational forces did not result in any increase in the rate of OTM.

## Materials and methods

### Trial design

This study was a parallel group two arm randomized controlled clinical trial with 1:1 allocation ratio that was reported following the CONSORT statement^38^. The study was approved by the Evidence Based Center, and the Research Ethics Committee and performed at the Faculty of Dentistry, Cairo University. The study is registered on clinicaltrial.gov with id NCT05818527 19/04/2023. All patients were acquainted with the study procedures, and signed informed consents. No changes or modifications were done to the original methodology of the research after trial commencement. All methods were performed in accordance with the CONSORT guidelines and regulations.

### Sample size calculation

The sample size for the current study was calculated based on the results of Kau et al.^11^. A total sample size of 52 canines was calculated to detect a large effect size (d = 0.8) with 80% power and 5% significance level. This number has been increased to a total sample size of 64 canines to count for the expected sample attrition. The outcome variable is normally distributed. The sample size was calculated using G-Power program (University of Düsseldorf, Düsseldorf, Germany).

### Participants, eligibility criteria, and settings

Patients who met the eligibility criteria (Table 8) were invited to participate in the study. All participants signed their informed consent. Subjects were randomly assigned to intervention (AcceleDent Aura) or no-appliance groups using computer randomization sequence generation (https://www.random.org/) with 1:1 allocation ratio (16 patients/group with 32 canines/group). No between-group differences were found in age. It was not possible to mask the patients or the orthodontist providing the treatment, however, the outcome assessor was masked to the intervention.

**Table 8:** Eligibility criteria for patients included in the study.

Inclusion criteria	Exclusion criteria
Female patients	Systemic disease or syndrome
Age 15–21 years	Abnormalities in teeth size and/or shape
Full permanent dentition	Vertical, transverse or antero-posterior skelet al discrepancies
Good general and oral health	History of previous orthodontic treatment
Severe crowding or protrusion requiring first premolars extractions	Anti-inflammatory medication

All subjects received pre-adjusted MBT 0.022 × 0.028-inch slot brackets (3M Gemini et al. brackets, 3M Unitek Corporation, Monrovia, A, USA) on their upper and lower arches excluding the upper first premolars. The upper arch wire sequence in the initial levelling and alignment phase was tailored according to the severity of crowding from 0.014-inch NiTi archwire, until reaching 0.016 × 0.022-inch stainless steel arch wire. Self-drilling miniscrews (TADs-Hubit, Korea), 1.8 × 8 mm were placed buccally perpendicular to the labial plate of bone at the mucogingival junction between the upper second premolar and first molar bilaterally. Indirect anchorage was prepared bilaterally by inserting a L-shaped 0.019 × 0.025-inch stainless-steel wire in the auxiliary tube of the upper first molar bands and fixed to the mini-screws with flowable composite.

### Interventions and outcomes

**Figure 2:**
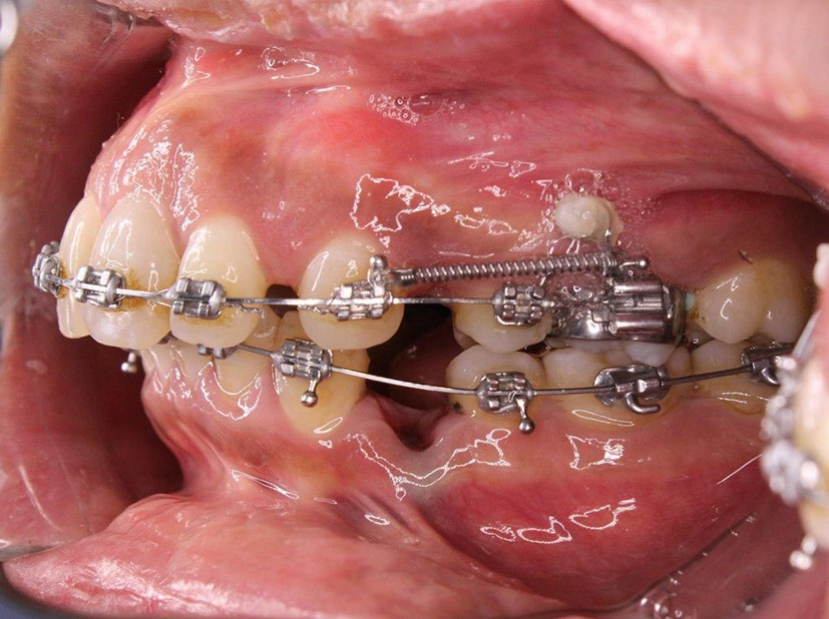
Intraoral photo showing the appliance and the canine retraction using NiTi coil spring delivering 150 gm.

At the end of the levelling and alignment phase, the patients were referred for upper bilateral first premolar extraction and an upper alginate impression was taken. Retraction of the canine was done using NiTi coil spring delivering a force of 150 gm per side calibrated using digital force gauge, attached between the hook of the canine bracket and the first molar tube on a of 16 × 22 stainless steel basal archwire (Figure 2). The intervention group subjects were given AcceleDent devices which delivered gentle micropulses (0.25 N at 30 Hz) and were instructed to wear them every day for 20 mins according to the manufacturer Acceledent protocol^®^ instructions (OrthoAccel Technologies Inc, Bellaire, TX, USA). The patient compliance to intervention instructions were monitored by asking the patient and writing every day on chart how long the patient used it and compare this with the data download from the appliance that recorded their daily usage during the period of space closure.

Follow-up visits were scheduled every four weeks. At each follow up visit, recalibration of the NiTi retraction spring was done using the same force gauge when necessary to maintain 150 gm force delivery. TADs stability and occlusal interferences during canine retraction were also regularly checked. An alginate impression for the upper arch was taken monthly.

**Figure 3:**
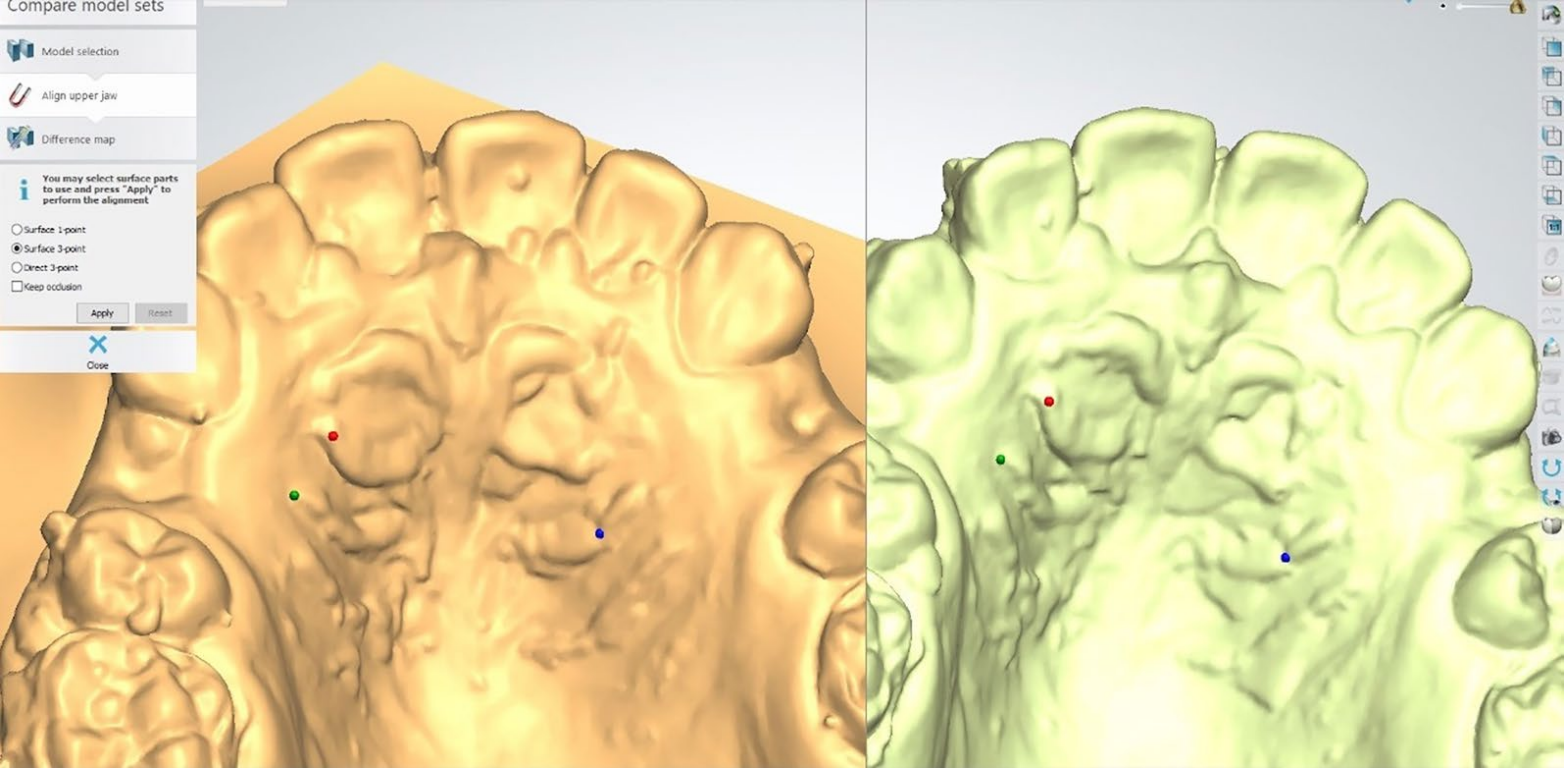
Localization of the medial point of the third rugae for superimposition of the successive models .

**Figure 4:**
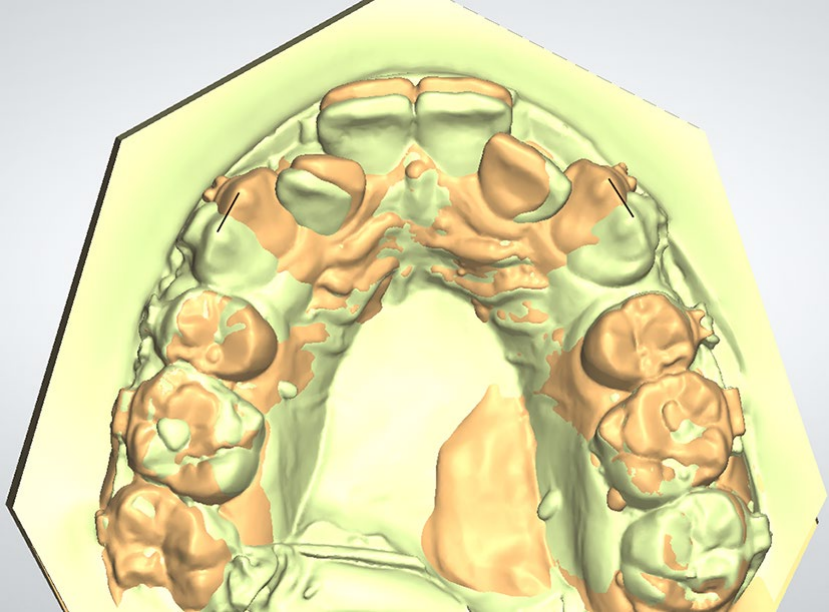
The superimposition of the successive models.

The plaster models collected (T0-T4) were digitized using desktop scanner (3Shape R500, 3shape, Copenhagen, Denmark) The canine retraction was assessed using two methods; the incremental rate of canine retraction and the total distance travelled by the canine. Using the 3Shape OrthoAnalyzer software (3Shape, Copenhagen, Denmark) the four consecutive models (T1–T4) were superimposed^39^ on the base model (T0) using three points registration upon the third rugae area (Fig. 3). Colour-mapped superimposition was used to verify the accuracy of the superimposition. The difference in the position of the canine cusp tip was used to calculate the incremental rate of canine retraction (Fig. 4). For intra- and inter-rater reliability, measurements of the digital models were done by the same operator (NA) twice, 2 weeks apart and repeated by another operator (MA).

### Cone beam computed tomography

Pre- and post-retraction CBCT images were obtained for each patient using the same CBCT machine with the following parameters: Resolution (Voxel size): 0.3\0.3 mm, exposure time: 10–20 s, Anode voltage: 57–90 kV, field of view (FOV): 6 × 8 cm limited to the maxilla, and anode current: 4–16 mA. A total of 36 CBCT images were obtained at the end of the study (18 pre-retraction and 18 post-retraction) upon which the analysis was done.

**Figure 5:**
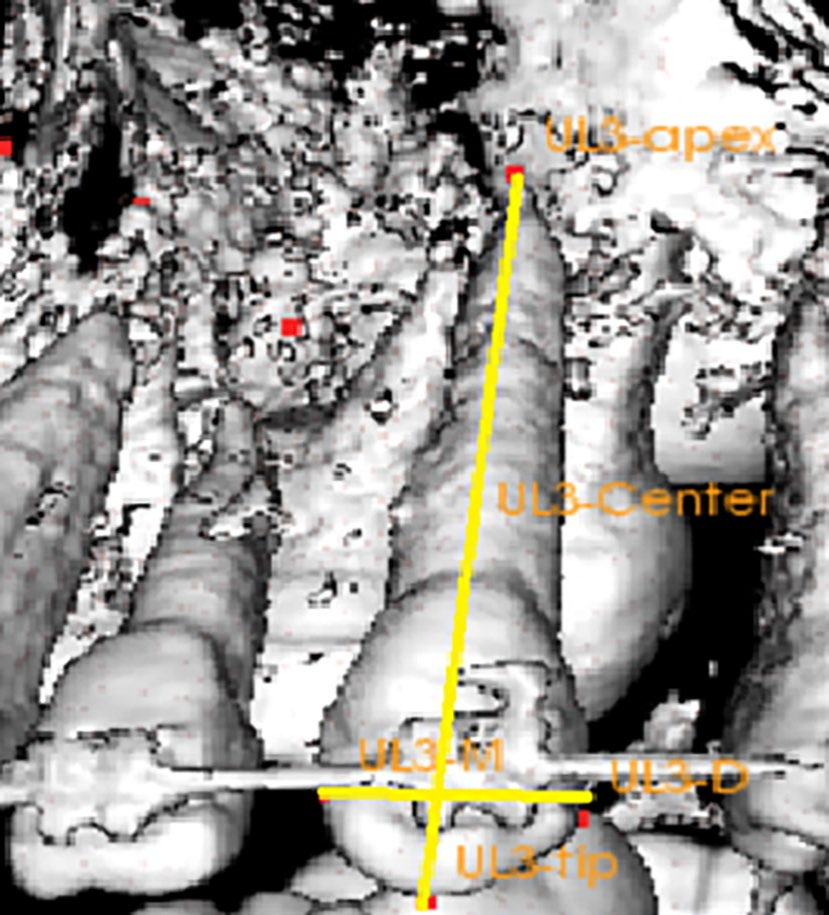
Volumetric views showing reference lines used for CBCT measurements; long axis and horizontal axis of maxillary canine.

The DICOM files obtained from the imaging centre were manipulated using In vivo 5 (Anatomage) version 5.3 software to perform the CBCT measurements as follows: Landmarks (Table 9), reference lines/planes (Table 10) (Fig. 5) and measurements (Table 11) were all recorded in their corresponding modules. Then, an analysis was created and saved to be used for all pre- & post-retraction CBCT images.

**Table 9:** Showing CBCT landmarks.

Landmark	Abbreviation	Definition
1- Anterior nasal spine	ANS	Most anterior point on the tip of the anterior nasal spine
2- Posterior nasal spine	PNS	Most posterior point on the hard palate at the tip of the post nasal spine
3- Incisive foramen	IF	The most posterior point at the opening of the incisive foramen at midline from the occlusal view
4- Maxillary Right canine cusp tip	UR3-tip	The most incisal point on the maxillary right canine cusp tip
5- Maxillary Right canine root apex	UR3-apex	The most apical point at the apex of maxillary right canine
6- Maxillary right canine center	UR3-C	The mid-point between the UR3-tip and UR3-apex on the maxillary right canine long axis
7- Maxillary left canine cusp tip	UL3- tip	The most incisal point on the maxillary left canine cusp tip
8- Maxillary left canine root apex	UL3- apex	The most apical point at the apex of maxillary left canine
9- Maxillary left canine center	UL3-C	The mid-point between the UL3-tip and UL3-apex on the maxillary left canine long axis
10- Maxillary right first molar mesiobuccal cusp tip	UR6MB-cusp	The point at the maxillary right mesiobuccal cusp tip
11- Maxillary right first molar Distobuccal cusp tip	UR6DB-cusp	The point at the maxillary right distobuccal cusp tip
12- Maxillary right first molar mesiobuccal root apex	UR6MB-apex	The point at the maxillary right mesiobuccal root apex
13- Maxillary right first molar center	UR6-C	The mid-point between the UR6MB-cusp and UR6MB-apex on the maxillary right first molar long axes
14- Maxillary left first molar mesiobuccal cusp tip	UL6MB-cusp	The point at the maxillary left mesiobuccal cusp tip
15- Maxillary left first molar distobuccal cusp tip	UL6DB-cusp	The point at the maxillary left distobuccal cusp tip
16- Maxillary left first molar mesiobuccal root apex	UL6MB-apex	The point at the maxillary left mesiobuccal root apex
17- Maxillary left molar center	UL6-C	The mid-point between the UL6MB-cusp and UL6MB-apex on the maxillary left first molar long axes

**Table 10:** CBCT Reference planes and lines.

Reference	Abbreviation	Definition
1- Mid-sagittal plane	MSP	Plan formed between points ANS, PNS and IF
2- Horizontal plane	HP	Plan formed between points ANS, PNS and perpendicular to MSP
3- Frontal plane	FP	Plan at IF and perpendicular to MSP and HP
4- Maxillary right canine long axis	UR3-LA	Line connecting UR3-tip with UR3-apex
5- Maxillary left canine long axis	UL3-LA	Line connecting UL3-tip with UL3-apex
6- Maxillary right first molar long axis	UR6-LA	Line connecting UR6MB-cusp with UR6MB-apex
7- Maxillary left first molar long axis	UL6-LA	Line connecting UL6MB-cusp with UL6MB-apex
8- Maxillary right first molar horizontal axis	UR6-HA	Line connecting UR6MB-cusp and UR6DB-cusp
9- Maxillary left first molar horizontal axis	UL6-HA	Line connecting UL6MB-cusp and UL6DB-cusp

**Table 11:** CBCT measurements.

Measurement	Abbreviation	Definition
a- Distance travelled and anchorage loss:		
1- Maxillary right canine cusp tip distance travelled	UR3-tip-dist	Distance between maxillary right canine cusp tip and Frontal Plane from sagittal view
2- Maxillary right canine center distance travelled	UR3-C-dist	Distance between maxillary right canine center and Frontal Plane from sagittal view
3- Maxillary right canine root apex distance travelled	UR3-apex-dist	Distance between maxillary right canine root apex and Frontal Plane from sagittal view
4- Maxillary left canine cusp tip distance travelled	UL3-tip-dist	Distance between maxillary left canine cusp tip and Frontal Plane from sagittal view
5- Maxillary left canine center distance travelled	UL3-C-dist	Distance between maxillary left canine center and Frontal Plane from sagittal view
6- Maxillary left canine root apex distance travelled	UL3-apex-dist	Distance between maxillary left canine root apex and Frontal Plane from sagittal view
7- Maxillary right first molar mesio-buccal cusp tip loss of anchorage	UR6-tip-AL	Distance between maxillary right first molar mesio-buccal cusp tip and Frontal Plane from sagittal view
8- Maxillary right first molar center loss of anchorage	UR6-C-AL	Distance between maxillary right first molar center and Frontal Plane from sagittal view
9- Maxillary right first molar mesio-buccal root apex loss of anchorage	UR6-apex-AL	Distance between maxillary right first molar mesio-buccal root apex and Frontal Plane from sagittal view
10- Maxillary left first molar mesio-buccal cusp tip loss of anchorage	UL6-tip-AL	Distance between maxillary left first molar mesio-buccal cusp tip and Frontal Plane from sagittal view
11- Maxillary left first molar center loss of anchorage	UL6-C-AL	Distance between maxillary left first molar center and Frontal Plane from sagittal view
12- Maxillary left first molar mesio-buccal root apex loss of anchorage	UL6-apex-AL	Distance between maxillary left first molar mesio-buccal root apex and Frontal Plane from sagittal view
b- Tipping:		
13- Maxillary right canine tipping to frontal plane	UR3-tipping-FP	Angle between maxillary right canine long axis and Frontal Plane from sagittal view
14- Maxillary right canine tipping to horizontal plane	UR3-tipping-HP	Angle between maxillary right canine long axis and Horizontal Plane from sagittal view
15- Maxillary left canine tipping to frontal plane	UL3-tipping-FP	Angle between maxillary left canine long axis and Frontal Plane from sagittal view
16- Maxillary left canine tipping to horizontal plane	UL3-tipping-HP	Angle between maxillary left canine long axis and Horizontal Plane from sagittal view
17- Maxillary right first molar tipping to frontal plane	UR6-tipping-FP	Angle between maxillary right first molar long axis and Frontal Plane from sagittal view
18- Maxillary right first molar tipping to horizontal plane	UR6-tipping-HP	Angle between maxillary right first molar long axis and Horizontal Plane from sagittal view
19- Maxillary left first molar tipping to frontal plane	UL6-tipping-FP	Angle between maxillary left first molar long axis and Frontal Plane from sagittal view
20- Maxillary left first molar tipping to horizontal plane	UL6-tipping-HP	Angle between maxillary left first molar long axis and Horizontal Plane from sagittal view
c- Torque:		
21- Maxillary right canine torque to mid-sagittal plane	UR3-torque-MSP	Angle between maxillary right canine long axis and mid-sagittal plane from frontal view
22- Maxillary right canine torque to horizontal plane	UR3-torque-HP	Angle between maxillary right canine long axis and Horizontal Plane from frontal view
23- Maxillary left canine torque to mid-sagittal plane	UL3-torque-MSP	Angle between maxillary left canine long axis and mid-sagittal plane from frontal view
24- Maxillary left canine torque to horizontal plane	UL3-torque-HP	Angle between maxillary left canine long axis and Horizontal Plane from frontal view
25- Maxillary right first molar torque to mid-sagittal plane	UR6-torque-MSP	Angle between maxillary right first molar long axis and mid-sagittal plane from frontal view
26- Maxillary right first molar torque to horizontal plane	UR6-torque-HP	Angle between maxillary right first molar long axis and Horizontal Plane from frontal view
27- Maxillary left first molar torque to mid-sagittal plane	UL6-torque-MSP	Angle between maxillary left first molar long axis and mid-sagittal plane from frontal view
28- Maxillary left first molar torque to horizontal plane	UL6-torque-HP	Angle between maxillary left first molar long axis and Horizontal Plane from frontal view
d- Rotation:		
29- Maxillary right canine rotation to id-sagittal plane	UR3-rot-MSP	Angle between the maxillary right canine horizontal axis and mid-sagittal plane from occlusal view
30- Maxillary right canine rotation to frontal plane	UR3-rot-FP	Angle between the maxillary right canine horizontal axis and Frontal Plane from occlusal view
31- Maxillary left canine rotation to mid-sagittal plane	UL3-rot-MSP	Angle between the maxillary left canine horizontal axis and mid-sagittal plane from occlusal view
32- Maxillary left canine rotation to frontal plane	UL3-rot-FP	Angle between the maxillary left canine horizontal axis and Frontal Plane from occlusal view
33- Maxillary right first molar rotation to mid-sagittal plane	UR6-rot-MSP	Angle between the maxillary right first molar horizontal axis and mid-sagittal plane from occlusal view
34- Maxillary right first molar rotation to frontal plane	UR6-rot-FP	Angle between the maxillary right first molar horizontal axis and Frontal Plane from occlusal view
35- Maxillary left first molar rotation to mid-sagittal plane	UL6-rot-MSP	Angle between the maxillary left first molar horizontal axis and mid-sagittal plane from occlusal view
36- Maxillary left first molar rotation to frontal plane	UL6-rot-FP	Angle between the maxillary left first molar horizontal axis and Frontal Plane from occlusal view

### Measurements used in the CBCT analysis

Measurements of the total distance of canine retraction, the canine tipping, torque and rotation were analysed by measuring the angles between the long axis lines for the canines and the three reference planes were measured to detect tipping and torque movements. Also, the angle between the horizontal line of the first molar and the sagittal plane to detect canine rotation.

For assessment of root resorption, the axial guided navigation method explained by Castro et al.^40^ and Schwartz et al.^41^ was used. Using the software In-vivo 5, version 5.3(Anatomage, Inc., Santa Clara, CA 95054, USA). The pre- & post-retraction CBCTs obtained for each patient were used to evaluate the effect of AcceleDent on root resorption, the linear length between the root apex and cusps tip was measured.

**Figure 6:**
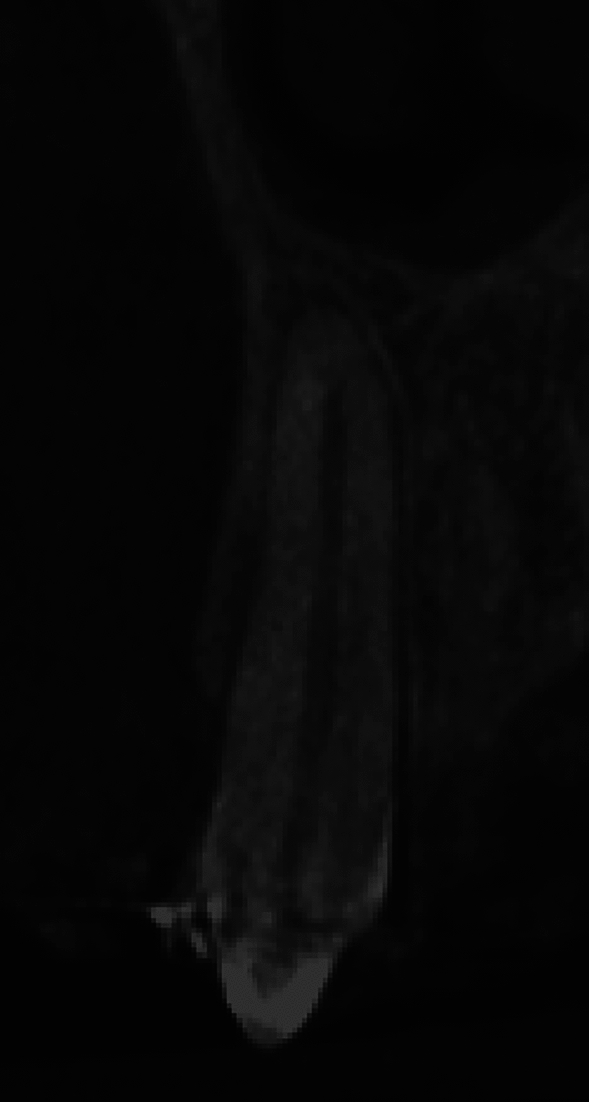
The CBCT image of the canine showing the canine root length.

The evaluation was carried by two blinded examiners. In order to fully visualize the root, the CBCT image was re-oriented on each root so that the cross-section would pass through the long axis of each canine (Fig. 6).

The degree of pain was measured by Visual Analog Score (VAS), these charts were filled by the patients for the following week after beginning of canine retraction at time intervals (0, 24 h , 48 h, 72 h and 7 days ) and were gathered at the end of the week. The pain VAS was in the format of a chart that contained a series of 10-cm horizontal scales on which the patient marked the degree of pain (0–10, where 0 refer to no pain and 10 refer to sever pain) at the indicated time periods.

### Statistical analysis

Data management and statistical analysis were done using Statistical Package for Social Sciences, Version 21.0 (SPSS Inc., Chicago III) for Windows. Shapiro–Wilk tests of normality were used to test normality of all quantitative variable distributions. Canine retraction in millimetres was presented as mean and standard deviation (SD). Kruskal–Wallis test was used to test the difference in the incremental rate of retraction between the two groups. Independent t-test was used to determine the statistical differences in the total distance travelled. *P*-value < 0.05 was considered statistically significant. All tests were two-sided. Concordance correlation coefficients (CCCs) were calculated to detect the intra- and inter-examiner reliability of the measurements.

## Conclusions

- This prospective randomized clinical trial showed no evidence that AcceleDent Aura appliance in conjunction with fixed orthodontic appliance had any effect on acceleration of the rate of canine retraction in the maxillary arch.
- Pain level couldn’t be reduced by vibrational force with AcceleDent device during orthodontic treatment.
- Root length was not affected by vibrational forces.

## Limitations

Patients were instructed to apply mechanical vibrations for 20 min /day for 4 months, thus patient cooperation might have affected the study’s outcome. There is nothing to stop an individual from simply turning on the device without placing it in his or her mouth, if one wishes to truly conceal noncompliance. Because there is a lack of long-term data on compliance with the AcceleDent appliance, a comparison cannot be made with other studies. Neither the participants nor the clinician were blinded to the appliance group but the assessor were. The study only investigated the rate of retraction of canines over a 16-week period, which did not represent the entire orthodontic treatment. A larger sample size was required to evaluate further the long-term effect of vibration on OTM and root resorption. There was no sham device, no blinding of the intervention.

### Supplementary Information

Supplementary Information 1.

Supplementary Information 2.

Supplementary Information 3.

## Data Availability

All data generated or analyzed during this study are included in this published article [and its supplementary information files].
